# Assessment of stanniocalcin-1 as a prognostic marker in human esophageal squamous cell carcinoma

**DOI:** 10.3892/or.2011.1607

**Published:** 2011-12-22

**Authors:** MITSUHIRO SHIRAKAWA, YOSHIYUKI FUJIWARA, YURIKA SUGITA, JEONG-HO MOON, SHUJI TAKIGUCHI, KIYOKAZU NAKAJIMA, HIROSHI MIYATA, MAKOTO YAMASAKI, MASAKI MORI, YUICHIRO DOKI

**Affiliations:** Department of Gastroenterological Surgery, Graduate School of Medicine, Osaka University, Suita, Osaka 565-0871, Japan

**Keywords:** stanniocalcin-1, esophageal carcinoma, HIF-1, p53

## Abstract

Stanniocalcin-1 (STC1) is a secreted glycoprotein hormone and highly expressed in various types of human malignancies. Although evidence points to the role of STC1 in human cancers, the clinical significance of STC1 expression in esophageal cancer has not been well established. Quantitative reverse transcriptase-polymerase chain reaction and immunohistochemistry were performed to assess the expression of STC1 in the cancer cell line TE8 and esophageal cancer tissues from 229 esophageal squamous cell carcinomas (ESCC). Surgically-resected tissue sections were immunostained for potential regulators of STC1 expression, hypoxia-inducible factor-1α (HIF-1α) and p53. Marked increase in STC1 mRNA and protein expression was noted in TE8 cells cultured under hypoxic conditions. Overexpression of STC1 mRNA was noted in ESCC tumors compared to normal counterparts. Positive immunohistochemical staining for STC1 protein was observed in 38.9% of patients, and correlated significantly with advanced pT status (P=0.019), poor prognosis [overall survival (P<0.0006) and disease-free survival (P<0.0002) of ESCC patients who had undergone curative surgery]. Positive staining for HIF-1α and p53 proteins in ESCC did not correlate with STC1 expression. The results showed marked induction of STC1 expression under hypoxia in cultured cells and in esophageal cancer cells and that overexpression of STC1 was an independent prognostic factor in patients with esophageal cancer who had undergone curative surgery. STC1 is a potentially useful biomarker for ESCC treatment.

## Introduction

Esophageal squamous cell carcinoma (ESCC) is one of the most lethal malignancies of the gastrointestinal tract (1,2). Although surgical resection is one of the most effective treatments for ESCC, it is often followed by recurrence, and hence ESCC is known to be associated with poor prognosis. Nevertheless, the implementation of a multidisciplinary approach in recent years such as chemotherapy, chemoradiotherapy, and surgery has improved the prognosis of ESCC (3,4). To select patients most suitable for the multidisciplinary treatment requires the use of a simple and accurate prognostic marker. Identifying suitable biomarkers to predict recurrence will probably be key to select suitable candidates for adjuvant therapy and improve prognosis.

Stanniocalcin-1 (STC1) is an anti-hypercalcemic hormone, which was discovered in the corpuscles of Stannius (an endocrine gland unique to bony fish) (5). In 1995, Chang *et al* reported the isolation of the human counterpart of STC1, which was highly homologous to the fish hormone (6). The human STC1 cDNA was cloned as a DNA fragment, whose expression level was different in SV40-transfected immortalized human fibroblast cells compared to mortal ones, indicating that STC1 might play a role in human cell immortalization (6). STC2, a STC1 paralog, was identified following searching of the expressed sequence tag (EST) databases for STC1-related sequences (7). Both STCs are expressed in a variety of human tissues, including endocrine glands and hormone responsive organs.

We previously reported a tendency for STC1 mRNA overexpression in hepatocellular carcinoma (HCC) and colorectal cancer compared with background cancer-free tissues, and also that STC1 mRNA might be a useful molecular marker for the detection of cancer cells in blood of patients with HCC (8). Furthermore, other investigators reported STC1 overexpression in breast adenocarcinoma and MEN2B medullary thyroid cancer (9). STC1 also appears to be involved in human carcinogenesis, including colon and breast cancers (10). Law *et al* (11) identified the binding motif of the hypoxia-inducible factor 1α (HIF-1α) in the promoter region of the STC1 gene, which might be responsive to hypoxia in human tumors. Furthermore, Law *et al* showed that a putative p53-responsive element was located at the transcription start site of the STC1 promoter region and that STC1 might be one of the target genes of the tumor suppressor, p53. Although there is growing evidence for the role of STC1 in human cancer, the clinical significance of STC1 overexpression in human cancer has not been established.

The present study was designed to determine the protein expression of STC1 in surgically resected ESCC specimens and its correlation with various clinical parameters, HIF-1α expression and p53 status.

## Materials and methods

### Patients and specimens

We obtained esophageal cancer tissues from 229 patients who underwent curative esophageal surgery at the Department of Gastroenterological Surgery, Osaka University Hospital during the period from 1998 to 2007. All tumors were confirmed to be ESCC by histopathological examination. The patients included 205 males and 24 females, aged between 36 and 85 (median, 63 years). Table I lists the patients’ characteristics. The pathological features of the specimens were classified based on the 6th edition of the TNM classification based on the International Union against Cancer (UICC). Of the 229 patients, 113 (49%) underwent neoadjuvant chemotherapy (NAC) followed by surgery. The regimen consisted of two courses of 5-fluorouracil, adriamycin, and cisplatin (FAP therapy) (12,13).

### Evaluation of clinical response to NAC

The clinical response to FAP therapy was evaluated for the main tumor and metastatic lymph nodes on enhanced chest and abdominal CT scans at 5-mm slices. Two CTs were obtained; one before the commencement of the first cycle of FAP therapy and another at the end of two FAP courses, about 2 weeks later. The response to NAC was defined as complete response (CR), partial response (PR), stable disease (SD), and progressive disease (PD) using the method described previously by our group (14,15). For simple statistical analysis, patients with CR and PR were grouped together as the responders, and those with SD and PD as the non-responders.

### Immunohistochemical analysis

The expression of STC1, HIF-1α and p53 proteins was evaluated by immunohistochemical (IHC) analysis using 4-mm thick sections of 10% formalin-fixed and paraffin-embedded tissue blocks. For IHC staining, the tissue slides were deparaffinized in xylene and then rehydrated through graded ethanol solutions. For antigen retrieval, these slides were incubated in 10 mM citrate buffer (pH 6.0) at 95°C for 40 min. Endogenous peroxidase activity was blocked with 0.3% hydrogen peroxide in methanol for 20 min. Non-specific binding was blocked with 10% normal serum for 20 min. Subsequently, the tissue slides were incubated overnight with the following antibodies at 4°C in a humidity chamber; STC1 antibody (sc-14346; 1:400 dilution; Santa Cruz Biotechnology, Santa Cruz, CA), HIF-1α antibody (NB100-105; 1:100 dilution; Novus Biologicals, Littleton, CO), and p53 antibody (DO-7; sc-47698; Santa Cruz Biotechnology). The sites of antibody binding were visualized with the ABC peroxidase detection system (Vector Laboratories, Peterborough, UK). Negative controls of immunohistochemical reactions were prepared by omitting the primary antibody (Fig. 2A). Positive staining of normal gastric glands was used as a positive control (Fig. 2B). The expression of STC1, HIF-1α, and p53 protein was considered negative when no cancer cells were stained immunohistochemically in all examined cancer cells, or otherwise as positive.

### Immunocytochemical analysis

TE8 cells, ESCC cells, were seeded in Lab-Tek II Chamber Slide System (Nalge, Nunc International, Rochester, NY). The cells were incubated under either normoxic or hypoxic conditions. After incubation, they were fixed at room temperature with 4% paraformaldehyde for 30 min and permeabilized with 1% NP-40 for 10 min. Later, the slides were treated with peroxidase blocking reagent (0.3% hydrogen peroxide in methanol) for 30 min at room temperature. The method used for staining STC1 protein was identical to that described above.

### Cell culture

TE8 cells were grown as a monolayer in RPMI-1640 medium (Sigma, St. Louis, MO) supplemented with 10% heat-inactivated fetal bovine serum and 1% penicillin-streptomycin (Pen Strep, Invitrogen, Carlsbad, CA) in 10-mm dishes. The cells were incubated in 5% CO_2_/95% air at 37°C. After incubation overnight, the cells were exposed to hypoxia for 6, 12, 24, or 48 h. To achieve cellular hypoxia, the cultures were maintained in an air-tight modular incubator chamber. The O_2_ content in the incubator chamber was maintained at about 1%.

### RNA extraction and reverse transcription

Crushed surgical specimens and cells of a cancer cell line were dissolved in TRIzol (Invitrogen). Total RNA was extracted using the method supplied by the manufacturer. Complementary DNA (cDNA) was generated from 1 mg RNA in a final volume of 20 mg, containing oligo-(dT)15 primer, avian myeloblastosis virus transcriptase, with a reverse transcription (RT) system (Promega, Madison, WI).

### Real-time quantitative RT-PCR analysis

The primer set 5′-TGA GGTCGTCCAGCTGCCCAATC-3′ (forward) and 5′-GGC ACAGTGGTCTGTCTGCAGGATG-3′ (reverse) was designed to amplify the fragments of STC1 cDNA for real-time quantitative RT-PCR analysis. The integrity of all RNA samples was verified by quantitative RT-PCR for porphobilinogen deaminase (PBGD) in each sample. The PCR conditions were set as follow: one cycle at 95°C for 10 min, then 40 cycles at 95°C for 15 sec and 60°C for 25 sec. The emission intensity of SYBR-Green was detected in real-time with the LightCycler 3.5 instrument (Roche Diagnostics, Mannheim, Germany). The external standards were prepared by serial dilution (1:1-1:100,000) of cDNA from the TE8 cell line. Quantitative RT-PCR was performed at least three times, including a no-sample control, as a negative control. The value of the STC1 expression was divided by that of the PBGD in each sample.

### Statistical analysis

Statistical analysis was performed with JMP^®^ software (JMP version 8.0.1, SAS Institute, Cary, NC). The associations of STC1 expression with various clinicopathological features were assessed by the χ^2^-test. Disease-free survival (DFS) and overall survival (OS) were assessed with the Kaplan-Meier method and compared by the log-rank test. All parameters found to be significant on univariate analysis were entered into a multivariate survival analysis using the Cox proportional hazard model. A P-value of <0.05 was considered to indicate significant differences.

## Results

### STC1 expression in esophageal cancer tissues

**Figure 1:**
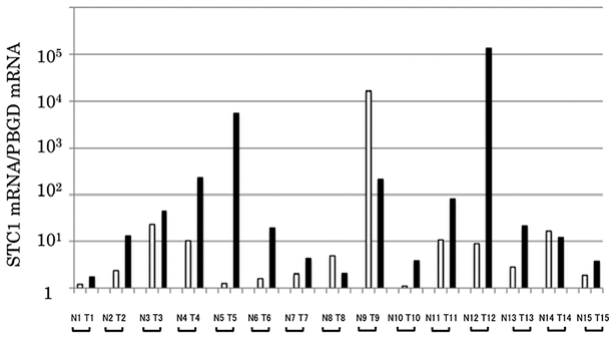
STC1 mRNA expression in esophageal normal and tumor tissues of 15 patients with esophageal cancer. N, normal tissue; T, tumor tissue.

**Figure 2:**
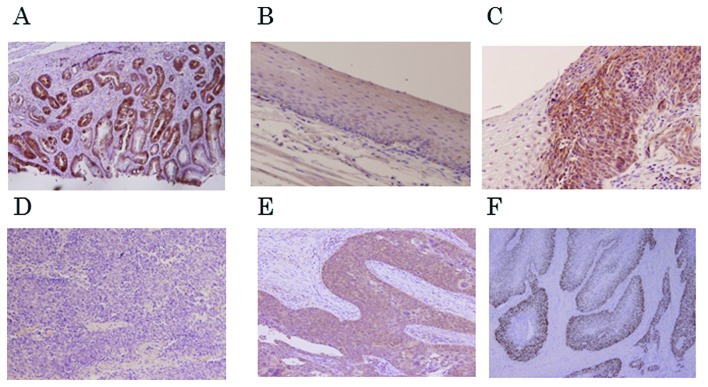
Immunohistochemical analysis of STC1 expression in esophageal cancer. (A) Normal squamous epithelium was negative for STC1 expression. (B) Normal gastric glands showed positive staining for STC1, which was used as a positive control. (C) Positive staining for STC1 in esophageal squamous cell carcinoma (ESCC). (D) Negative staining for STC1 in ESCC. (E) Positive staining for HIF-1α in ESCC. (F) Positive staining for p53 in ESCC. Magnification, ×100.

We evaluated the STC1 mRNA expression levels in freshly-resected human tissues from 15 patients who had undergone esophageal surgery. Twelve cases out of 15 showed higher expression of STC1 mRNA in the tumor tissue compared with normal counterparts (Fig. 1). Furthermore, examination of STC1 expression in 229 cases with esophageal cancer by immunohistochemistry, showed positive staining in 89 cases (38.9%), at least in part of the tumor, and the staining was mainly localized in the cytoplasm of tumor cells (Fig. 2C). In the remaining 140 cases (61.1%), staining for STC1 was negative throughout the tumor tissue (Fig. 2D). On the other hand, staining was negative in the normal esophageal epithelium (Fig. 2A). The STC1-positive cells were localized in various parts of the tumor including tumor surface, central zone, and deepest areas of the esophageal wall (Fig. 2E and F).

### Correlations between STC1 expression and clinicopathological parameters

Table I shows the correlation between STC1 expression detected by immunohistochemistry and various clinicopathological parameters in 229 patients with esophageal cancer. The proportion of STC1-positive cases was significantly higher in advanced pathological T stage (pT 3-4) than the other T stages (pT 1-2) (44.5 vs. 28.9%, respectively, P=0.019). On the other hand, other parameters listed in Table I (age, gender, tumor location, number of metastatic lymph nodes, ly status, v status, pStage and with or without NAC) did not correlate with STC1 expression.

### Prognostic significance of STC1 expression in ESCC

**Figure 3:**
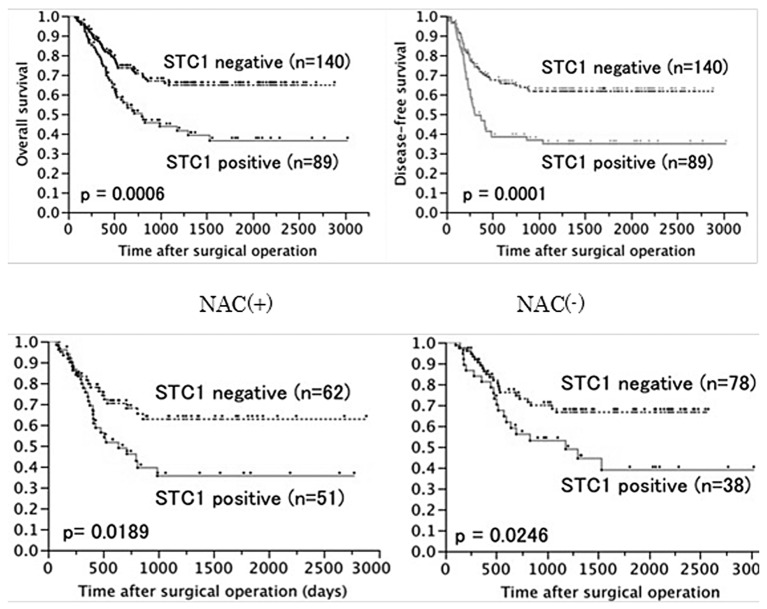
Survival rates according to STC1 expression in patients with esophageal cancer. The overall survival rate (A) and disese-free survival rate (B) classified by STC1 expression were plotted by the Kaplan-Meier method. Differences between the two groups (STC1-positive and STC1-negative) were evaluated by the log rank test. The STC1-positive group showed siginificantly poorer prognosis for both overall survival and disease-free survival than the STC1-negative group. (C) Overall survival rates of patients who received NAC according to STC1 expression. (D) Overall survival rates for patients treated by surgery alone according to STC1 expression.

Patients with STC1-positive tumors had significantly poorer overall survival (OS) and disease-free survival (DFS) than those with STC1-negative tumors (5-year OS, 36.4 vs. 64.7%, P=0.0006; 5-year DFS, 35.0 vs. 61.0%, P=0.0001, Fig. 3A and B). STC1 expression was also a poor prognostic factor in 113 patients who received NAC (P=0.0189, Fig. 3C) and 116 patients who did not (P=0.0246, Fig. 3D). Univariate analysis showed that histological type, pT stage, number of pathologically positive lymph nodes (number of pN), lymphatic invasion (ly), venous invasion (v), and STC1 expression correlated significantly with poor overall survival (Table II). These six parameters were then entered into multivariate analysis. The results identified pT, number of pN, ly and STC1 expression as independent and significant prognostic factors (Table II).

### STC1 expression and clinical response to chemotherapy

The clinical response was evaluated in 110 patients out of the 113 patients who received NAC by comparing CT images performed before and after chemotherapy. Sixty-two patients (56%) were defined as responders and 48 (44%) were non-responders. There was no relationship between clinical response to NAC and STC1 expression (data not shown).

### Effects of hypoxia on STC1 expression in esophageal cancer cell line (TE8)

**Figure 4:**
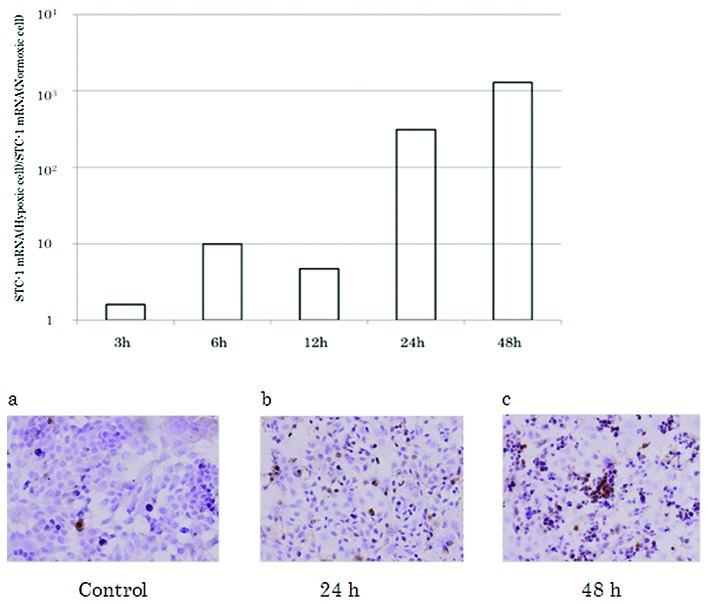
STC1 expression in TE8 cells. (A) STC1 mRNA expression in TE8 cells induced by culture under hypoxic condition for 3, 6, 12, 24, and 48 h. (B) STC1 protein expression examined by immunocytochemistry in TE8 cells cultured under normoxic conditions (a) and hypoxic conditions for (b) 24 and (c) 48 h.

Using quantitative RT-PCR, the STC1 mRNA expression level increased in a time-dependent manner in esophageal cancer cells (TE8) cultured under hypoxia (Fig. 4A); and at 48 h, the expression level was 1000 times that under normoxia. The time-dependent increase in STC1 protein expression in TE8 cells was also confirmed by immunocytochemistry (Fig. 4B).

### Expression of HIF-1a and p53 in ESCC tumors

Finally, we examined the expression of HIF-1α and p53 proteins by immunohistochemistry. Of the 229 tumor, 91 (40%) were positive for HIF-1α protein (Fig. 1E) and 177 (77%) for p53 expression (Fig. 1F). HIF-1α expression levels correlated significantly with advanced pT status, whereas p53 expression levels did not correlate with any of the clinicopathological parameters (data not shown). On the other hand, there were no significant correlations between HIF-1α and p53 expression in ESCC and with any of the clinicopathological features except with advanced pT status (data not shown). Furthermore, there were no significant correlations between each of HIF-1α and p53 protein expression and prognosis in ESCC patients (data not shown). To assess the relationship between STC1 expression and its potential regulators, HIF-1α and p53, we compared the expression patterns of these three proteins by immunohistochemistry in 229 ESCC tumors. The results showed neither a correlation between STC1 and HIF-1α expression nor between STC1 and p53 expression in esophageal tumors (Table III).

## Discussion

In the present study, we first showed that overexpression of STC1 protein was associated with tumor progression (advanced pT status) and was an independent prognostic factor for OS and DFS in surgically-resected specimens of ESCC. We previously reported that STC1 was expressed in various human cancer cell lines as well as in human colorectal, breast, stomach, esophageal, biliary tract, and liver tumors (8). Furthermore, we reported enhanced expression of STC1 in tumor tissues compared to the background normal tissues. The expression of STC1 mRNA has been widely investigated as a biomarker for cancer dissemination in HCC, breast cancer, and melanoma (8,16,17). Recent studies also stressed on the importance of STC1 in carcinogenesis (18). Although, the STC1 receptor(s) has not been discovered yet, McCudden *et al* (19) indicated that the mitochondrion is the cellular target of STC1 in their experiments using electron microscopy and receptor binding assays. Furthermore, STC1 was identified as one of the target genes in hypoxia and was demonstrated to be a stimulator of mitochondrial respiration (18,19). Based on these results, STC1 expression is thought to be related to the Warburg effect, in which HIF-1α plays a key role in modulating glycolytic enzymes and reprogramming of tumor metabolism (20,21). In this regard, Yeung *et al* (22) demonstrated the involvement of HIF-1α in the regulation of STC1 expression in nasopharyngeal cancer cell lines. Furthermore, Law *et al* (11) identified the HIF-1α binding motif in STC1 gene promoter and indicated that HIF-1α-mediated STC1 expression involves direct binding of HIF-1α to the STC1 promoter region. Based on the above background, we examined HIF-1α expression in the same set of ESCC tumors and assessed the correlation between STC1 and HIF-1α protein expression. About 40% of ESCC tumors were positive for HIF-1α expression, and the latter correlated with advanced pT status. However, our results showed no significant correlation between HIF-1α and STC1 expression. This may be due to the fact that the increased expression of HIF-1α under hypoxic conditions, such as the case in solid tumors, is mainly achieved by regulating the protein level through inactivation of the ubiquitin-proteasome system (23).

The DNA sequence of the STC1 promoter indicates one putative p53-responsive element located around the transcription start site. Lai *et al* (24) provided evidence for the p53-based regulation of STC1 expression in human cancer cells. In the present study, about 77% of ESCC tumors were positive for p53 expression but the latter did not correlate with poor OS or DFS in ESCC patients. Furthermore, there was no significant correlation between p53 protein expression and STC1 in ESCC tumors. It is possible that the positive immunohistochemical staining for p53 represents contamination of p53 protein overexpression and stabilization of mutated p53 protein, which could be the cause of no direct correlation between p53 and STC1. Further studies are needed to clarify the role of p53 in the regulation of STC1 in solid tumors.

The present results showed tissue hypoxia marked increased STC1 mRNA and protein expression. The hypoxic tumor microenvironment is considered to play a prominent role in the induction of chemoresistance (25). The mechanisms of hypoxia-induced chemoresistance include induction of the multidrug resistance (MDR) gene (26) activation of the glutathione system (27), Akt pathway (28), and induction of HIF-1α and its downstream molecules, such as MDR1 and vascular endothelial growth factor (VEGF) (29,30). In this study, we examined the association between STC1 expression and chemoresistance in patients provided with chemotherapy before surgery. However, the results showed no relationship between STC1 expression and chemoresistance.

Kita *et al* (31) observed that STC2, a paralog of STC1, was abundantly expressed in esophageal cancer and metastatic lymph nodes using microarray gene expression analysis. They also reported that STC2 mRNA expression in the same tumor correlated with various clinicopathological factors such as lymph node metastasis, distant metastasis, lymphatic invasion and stage classification. STC2 is also known as a HIF-1α target gene (32). However, the results showed no significant association between STC2 and HIF-1α mRNA expression. Further studies are needed to elucidate the exact roles of STC1 and STC2 in hypoxia and in progression of esophageal cancer.

In conclusion, the expression of STC1 determined by immunohistochemistry could be a useful predictor of poor prognosis of ESCC patients after surgical resection. Furthermore, the expression of STC1 in ESCC could be potentially useful for selection of ESCC patients most suited for a multidisciplinary therapeutic approach.

## Figures and Tables

**Table I:** Correlation between STC1 expression and various clinicopathological parameters.

Characteristics	STC1 positive, n	STC1 negative, n	P-value
Total	89	140	
Age, years^a^	62.5 (38–81)	64.1 (36–85)	0.709
Gender			
Male	78	127	
Female	11	13	0.463
Tumor location			
Ce/Ut	12	19	
Mt/Lt/Ae	77	121	0.985
T status			
pT 0-2	24	59	
pT 3-4	65	81	0.019
Number of pN			
<4	55	99	
≥4	34	41	0.162
ly status			
ly0	12	29	
ly1	35	51	
ly2-3	42	60	0.368
v status			
v0	48	77	
v1	24	42	
v2-3	17	21	0.694
pStage			
pStage 1-2	37	66	
pStage 3-4	52	74	0.408
Neo-adjuvant chemotherapy			
Yes	51	62	
No	38	78	0.329

a Data are average and (range).

Ce, cervical; Ut, upper thoracic; Mt, middle thoracic; Lt, lower thoracic; Ae, abdominal/esophageal; pT, pN, pStage, pathological classification; number of pN, the number of metastasis-positive lymph nodes; ly, lymphatic invasion; v, venous invasion.

**Table II:** Results of univariate and multivariate survival analyses for overall survival by the Cox proportional hazard model.

	n	HR	95% CI	P-value
Univariate survival analysis				
Age, <65/>65 years	103/126	1.380	0.736–1.693	0.195
Gender, male/female	205/24	1.008	0.435–1.834	0.982
Histopathology (poor, mod)/(well, other)	176/53	1.715	0.970–3.053	0.044
Location (Ce, Ut)/(Mt, Lt, Ae)	31/198	1.433	0.422–1.280	0.209
pT (T1, T2)/(T3, T4)	82/147	3.383	0.715–1.781	<0.001
Number of pN, ≥4/<4	154/75	2.978	1.273–3.134	<0.001
ly (ly0)/(ly1, ly2, ly3)	41/188	8.391	1.380–3.892	<0.001
v (v0)/(v1, v2, v3)	104/125	1.705	0.671–1.724	0.011
STC1 expression, positive/negative	140/89	2.039	1.115–2.635	0.001
Neo-adjuvant chemotherapy, yes/no	113/116	1.402	0.732–1.747	0.109
Multivariate survival analysis				
Histopathology (poor, mod)/(well, other)	176/53	1.6735	1.088–3.269	0.0621
pT (T1, T2)/(T3, T4)	82/147	2.1551	1.325–4.186	0.0048
Number of pN, ≥4/<4	154/75	2.0625	1.252–3.040	0.0012
ly (ly0)/(ly1, ly2, ly3)	41/188	4.9324	1.378–3.884	0.0009
v (v0)/(v1, v2, v3)	104/125	1.0340	0.677–1.720	0.8788
STC1 expression, positive/negative	140/89	1.6841	1.116–2.620	0.0162

HR, hazard ratio; CI, confidence interval; poor, mod, well, poorly, moderately and well differentiated squamous cell carcinomas, respectively; other, other types of carcinoma; Ce, cervical; Ut/Mt, upper thoracic/middle thoracic; Lt, lower thoracic; Ae, abdominal/esophageal; pT, pN, pStage, pathological classification; number of pN, the number of metastasis-positive lymph nodes; ly, lymphatic invasion; v, venous invasion.

**Table III:** Correlations between STC1, and HIF1α or p53 protein expression examined by immunohistochemical analysis.

	STC1			
			
	Positive	Negative	Total	P-value
HIF-1α				
Positive	40	51	91	
Negative	49	89	138	0.2146
p53				
Positive	72	105	177	
Negative	17	35	52	0.3344
Total	89	140	229	
